# A Women's Volunteer Reserve

**Published:** 1914-12-19

**Authors:** 


### A WOMEN'S VOLUNTEER RESERVE.

Apposite to the misconception of the Women's Voluntary Hospital Brigade is the announcement of a "Women's Volunteer Reserve," the members of which, we are told, are "sedulously training" in drill and rifle-shooting with a view "to defend" in event of invasion "other women, children, and infirm persons." The movement, curiously enough, appears to have been accorded the approval of some notable persons, and a meeting at the Mansion House has taken place. To believe that this movement has originated in pure, if overwrought, patriotism would be easier were the names grouped about it less prominently identified with the suffrage agitation and the wider feminism which aims at annihilating sex limitations generally. As it is, the suspicion of an *arrière-pensée* cannot be dismissed. The demand for votes for women is to be strengthened by references to women's prowess in the battlefield, and the fact that they have worn martial uniform and learned to let off a rifle without harm to themselves is to demolish one fundamental if commonplace objection to their political aspirations—viz., that the ultimate result of authority is force, and in all civilised communities force is reserved for masculine employment.

But apart from the suggestion of a lack of candour, what claim has the proposal to respect when considered upon its merits? To our mind it is at once dangerous and ludicrous. We may safely assume that no English General would consent to be encumbered with women warriors. If he did, what would be the effect upon the enemy? Assuming the invasion of these shores to have taken place, and that women then succeeded in killing some Germans, what is likely to be their own fate or the fate of their sex wherever the enemy gained the upper hand? The answer to this question is not in doubt. It is contained in the notice, among others, issued by the German commanders when at the outset of the war Belgium's neutrality was first violated. The inhabitants of a frontier town, Hasselt, were officially informed that "in case of civilians shooting on the German Army a third of the population [? male] will be shot." Even with combatants more restrained in their methods than our present enemies, the measures taken to prevent civilian attack are remorseless. If women took part in the fighting we may be sure every check or serious defeat of our troops would be followed by a massacre of their sex.

When we come to consider the subject sentimentally, and sentiment in these matters is powerful, what is more likely to deaden inspiration in a man fighting to defend his country and his home than the picture of women playing at soldiers, each personally ready to dabble in blood and make herself as familiar as she is able to be with horrors to save her from which he is asserting his manhood's right to die?

Readers of war news have been sickened by narratives of women and children in captured Belgian villages forced by the Germans to walk in front of their soldiers advancing to the attack. The originators of this movement are asking Englishmen to employ their womenkind to cover their rear in defence. What the answer of the nation will be ought not to be in question. Let the abnormal women who would attempt these things reflect. There is not even the poor excuse that our last line of defence has been reached. Happily, we learn that arrangements are in progress which will enable millions of men to be added to the numbers available for home defence. There is no lack of opportunity for women's help in many directions, but the men must do their own fighting. That few women are capable of becoming Amazons is so much the better for the world. Woman's ineffable function and privilege is to help to mitigate the horrors of war, not to add to them.

